# The muslim patient and medical treatments based on porcine ingredients

**DOI:** 10.1186/s12910-023-00975-0

**Published:** 2023-10-27

**Authors:** Ya’arit Bokek-Cohen, Limor D. Gonen, Mahdi Tarabeih

**Affiliations:** 1 Tel Aviv University, 30 Haim Levanon Street, Tel Aviv Postal code, Tel Aviv, 6997801 Israel; 2 Ariel University, 1 HaZionut St, Ariel, Israel; 3 The Academic College of Tel-Aviv-Yaffo, 2 Rabenu Yerucham St., Tel Aviv Postal code, Tel Aviv, 6161001 Israel

**Keywords:** Cultural competence, Muslim, Porcine-derived products, Religion, Xenotransplantation

## Abstract

Porcine-derived products serve as an effective solution for a wide range of human ailments; however, there may be objections to their use due to Islamic religious prohibitions on consuming products derived from pigs. In order to enhance the cultural competence of medical practitioners who treat Muslim individuals, which constitute about one fifth of the world population, this study aimed at evaluating the knowledge and positions of Muslim patients on this subject. A questionnaire presenting 15 uses of porcine-derived materials was filled out by 809 Muslims. The level of knowledge about the permissibility of these uses and participants’ position on whether it should be approved was assessed. Findings show that Muslims are not familiar with Islamic religious jurisprudence that permits the use of porcine products to save lives after it has undergone an essential transformation known as *Istihala*. The respondents expressed a negative attitude towards the medical use of porcine-derived substances based on imprecise knowledge about the permissibility of use of porcine-derived materials, devices and treatments. We offer recommendations for improving the informed consent obtained from Muslim patients prior to conducting porcine-based treatments.

**Supplementary Information:**

The online version contains supplementary material available at 10.1186/s12910-023-00975-0.

## Introduction

There are 1.6 billion Muslims in the world, representing 23% of an estimated 2023 world population of 8.1 billion [1]. Many of them live in Western countries and constitute an ethnic minority to which medical treatment should be tailored, according to their beliefs and values. In view of the increasing importance of patient-centered care around the world, this article focuses on the need to tailor medical care to Muslim patients when the medications, medical devices, or implants are derived from pigs, an animal considered impure according to Islam [2, 3]. There is little empirical data exploring the knowledge and attitudes of Muslim patients toward using such agents. Due to the progress of clinical trials in using porcine-derived products in the present and future, it is important to examine the theological views of Islam regarding this issue, to explore how Islam jurisprudence addresses the importance of human health, and the use of pigs for medical purposes [4].

Islam is an all-encompassing religion, which adresses all aspects of human life and takes care of its followers in terms of their safety in daily life, especially in situations that may be detrimental [5]. The revealed sources of Shariah are the Quran and Sunnah. Shariah is the legal practice derived from the teachings of the Quran, Islam’s sacred scripture, and Sunnah are the teachings of the Prophet Muhammad. Fiqh represents the human understanding and practices of the Shariah. In Islamic teaching, Shariah is the source of Muslim existence, as it represents the proper way of conducting one’s life as determined by God. The Shariah not only separates actions into required and forbidden, but also into the intermediate categories of recommended, discouraged, and permitted. Two studies that examine Islam in more depth show that maintaining life and good health is an important principle in the religion [6, 7]. Muslims are expected to maintain their physical health in order to fulfill their obligation to God. Followers of Islam are encouraged to treat themselves with medicine but to refrain from using anything that is not lawful under the Islamic Shariah [8].

In Islam, the prohibition against consuming porcine products is steeped in religious teachings. The Quran states that “you are forbidden [the consumption] of carrion, blood, swine flesh … for these are impure.” (Quran 5:3, 6:145). Life is sacred and has great value in Islam; therefore, it is considered a duty to save life [9] Islam teaches that the saving of life is paramount and should be held above all other religious beliefs [10]. Allah Almighty states that “… necessities overrule prohibitions,” and although the pig is specifically prohibited for consumption, it is permitted in certain situations where no lawful alternative exists. Human consumption of porcine-derived material and its permissibility in Islam can be traced back in the Quran; There are four verses in the Quran where it was clearly stated that eating the flesh of swine is forbidden (Surah Al Maeeda, Al-Qur’an, 5:3). “Forbidden to you (to eat) : dead meat, blood, the flesh of swine, and that on which hath been invoked the name of other than Allah;…(Surah Al Maeeda, Al-Qur’an ,5:3). “He hath only forbidden you dead meat, and blood, and the flesh of swine, and that on which any other name hath been invoked besides that of Allah. But if one is forced by necessity, without willful disobedience, nor transgressing due limits, - then he is guiltless. For Allah is Oft-forgiving Most Merciful”. (Surah Al-Baqarah, Al-Qur’an, 2:173). “He has only forbidden you dead meat and blood and the flesh of swine and any (food) over which the name of other than Allah has been invoked. But if one is forced by necessity, without willful disobedience, nor transgressing due limits, - then Allah is Oft-forgiving Most Merciful.”(Surah Al-Nahl, Al- Qur’an, 16:115). Say “I find not in the message received by me by inspiration any (meat) forbidden to be eaten by one who wishes to eat it, unless it be dead meat, or blood poured forth, or the flesh of swine, - for it is an abomination – or, what is impious, (meat) on which a name has been invoked, other than Allah’s. But (even so), if a person is forced by necessity, without willful disobedience, nor transgressing due limits, - thy Lord is Oft-forgiving. Most Merciful” (Sura-Al-Anaam, Al-Qur’an, 6:145).

Materials produced from pigs are used to deliver effective solutions for the treatment and prevention of a wide range of diseases and medical problems. In the study by Paris et al. [4] on the willingness of people to accept products, medications, and implants from a pig, demographic factors were found to affect this willingness, such as religion, age, gender, and education, and that there is a positive correlation between religious knowledge and positive attitudes toward use of porcine materials for these purposes. Transplantation of organs from a pig is the only effective treatment for organ failure at an early stage, since there is a severe shortage of human organs for transplantation [4]. Ingestion of medications containing inert ingredients derived from forbidden sources may offend the followers of Islam. Recommending these drugs to patients with religious prohibitions about consumption of porcine products may raise an ethical dilemma.

According to the Physician’s Desk Reference, 336 drugs on the list contain gelatin, while 756 drugs contain stearic acid as an inert chemical ingredient. When the gelatin and stearic acid in these drugs are derived from beef and/or pork products, adherents of Judaism, Islam, Hinduism, Buddhism, and Orthodox Christianity regard taking these drugs as an act that contravenes their religion and even as a sin [10]. Some patients refuse to receive medical treatment based on their religious beliefs, even though most major religions do not prohibit their followers from taking drugs whose capsule is composed of animal materials [11]. However, followers of certain religions may not be aware that the medicines they need to take contain ingredients that are forbidden to them. In a study of physicians by Sattar et al. 68% of the participants were unaware that 1000 drugs contained gelatin and/or stearic acid most commonly derived from pork products [12]. The study data suggest that although most patients were unaware of the presence of pork products in their medications, they identified it as an important issue that could affect their willingness to comply with treatment guidelines when they recognize that their physician respects their religion. There are reports of patients who stopped medication due to learning of the presence of porcine-derived ingredients in their medications [12]. This suggests that patients consider it important to be aware of the components of the drug before the doctor chooses the recommended medication.

The present article addresses a wide range of medical treatments using porcine-derived ingredients. Porcine-derived ingredients are commonly used in the following medications and treatments: Anticoagulants, e.g.,Warfarin, to prevent or treat blood clots; Pain medications; Antithrombotics [12]; Digestive supplements and cholelitholytics; Respiratory agents-treatments to help the lungs of pre-term babies develop ; Herbal gastrointestinal preparations; Immunoglobulin; Rabies immune globulin (human); Vaccine MMR vax PRO (a type of measles, mumps and rubella vaccine); Haemostatic agent [13, 14]; Digestive supplements [15]; some insulins ; Gelatin capsules; Surfactants - Poractant alpha; Hormones; Collagens ; Lipid emulsion-containing medications [16]; Porcine mesh implants in the repair of abdominal and skin transplants after burns [5]; biological products for knee arthroscopy [16] bioprosthetic heart valve (BHV) replacement [17]; Pig skin xenografts for burn treatment [19]; Xenotransplantation of pig chondrocytes [20].

A full understanding of the beliefs and practices of Muslim traditions is necessary in order to fully prepare and implement clinical trials, such as practicing on pigs for medical experiments or invasive operations in order to improve medical techniques and perform pig organ transplants. Therefore, if pig organs are to be used, it is important to consider the position of religious and ethnic groups on xenotransplantation using pig organs [21–25] Thus, for example, from the point of view of Islamic religious law regarding xenotransplantation from pig organs, we must consider the moral status of the act along with its ultimate purpose, the source of the transplanted organs, the implications of the act for the patient, and the social considerations involved in the practice [4].

There are many patients who currently need a kidney transplant and do not receive this treatment because of the severe shortage of human organ donors (Author, Co-author B, 2021, 2022). Xenotransplantation using pig organs may provide the most immediate solution to the shortage of human organ donors. Pig organs are the closest to human organs, and clinical trials with the pig show high rates of success [26–30]. The Muslim faith permits eating the flesh of pigs and using of porcine-derived surgical products in critical situations, termed “darrurah”, after all other alternatives have been exhausted: “dire necessity renders the impermissible to be permissible” [8]. So that what is normally not permitted is made permissible for saving a life and for healthcare purposes. Islamic clerics have ruled that pork can be used to save lives if it has undergone a transformation as declared in Fatwa number 6783 [31, 32], which says.

that dire necessity renders the impermissible to be permissible. A second condition is a situation called Istihala. IIstihala is a procedure that changes the nature of the defiled or forbidden substance to produce a different substance in name, properties, and characteristics. Istihala can be divided into three types. First, Istihala includes the transformation of physical appearance, secondly, transformation of chemical substances, and thirdly, the transformation both in physical and chemical composition. Physical transformation includes odor, taste, and color, while chemical transformation means the change of chemical substances in the materials. The physical and chemical changing of a substance together involves total transformation, hence, produces new materials [33, 34].

A conference of the Islamic Organization for Medical Sciences (IOMS) held in Kuwait in 1995 which was attended by over one hundred Islamic jurisprudents and eminent scholars ruled that “Transformation, which refers to converting one substance into another that differs in properties has the capacity to change substances that under Shariah are regarded as impure substances or found in an impure environment, into pure substances, and substances that are prohibited into permissible substances”. Thus, gelatin which is produced from the transformation of the bones, skin, and tendons of an impure animal becomes pure and is permitted for consumption” [33–35]. However, another group of Muslim jurists ruled that the pig is “rijs” or “najas al-ayn”, i.e., meaning essentially filthy. Therefore, every part of it – its flesh, hair, bones, and skin – are all considered impure and may not be used for any purpose except in a life-threatening situation when there is no other alternative [6]. An example of a situation that represents saving a life is reported by Gunardi: “A postpartum patient after a cesarean delivery needs treatment to dilute her blood. There is no other safe drug that can be used to dilute the blood other than Clexane, even though it contains an ingredient prohibited under Islam and if the condition of the patient is critical, then the use of the medication is necessary to save the patient“ [^4^, p. 30] Similarly, in the absence of any other effective treatment for diabetes, it is permissible to inject insulin derived from a pig and it is permissible to give a drug that contains alcohol if there is no substitute and it is essential for treating the disease [36] However, it is noteworthy that Muslims may follow different religious opinions and authorities on a given issue, and some Muslims may not be convinced, even by the fatwas and by local imams, that porcine products are permissible. They may put demands on the medical staff to prove that all the alternatives have been exhausted. There may even be differences of opinion on the matter between the patient and their family members. In cases where the patient is a child, then their parents decide for them, and in cases of disagreement between the mother and the father, they may turn to the Muslim hospital chaplain or the imam [37–39].

### Research objective / research questions

Muslim patients may experience distress when the attending physician recommends a treatment or medicinal product derived from pigs, due to their fear of violating the religious prohibitions of the Shariah. Despite the importance of this issue, there are still no empirical data that examined the knowledge of Muslims about whether these treatments are permissible, nor what is their approach to the question of whether religious authorities should allow the use of these substances. The present study is intended to fill this gap and describe the results of a large-scale research project conducted on this topic in Israel, where 21% of the country’s citizens are Muslims. We therefore specifically seek to answer the following research questions:

1. What is the level of knowledge among Muslims regarding the use of porcine-derived substances for medical purposes?
2. What do Muslims think about whether the use of porcine-derived substances should be permitted for medical purposes?
3. Is knowledge regarding the use of porcine-derived substances for medical purposes positively correlated with a positive attitude towards it?

We first describe the methodological aspects of the study, afterwards we present the results of the survey we conducted, and finally we discuss the significance of these results and their practical implications.

## Method

### Participants

The target population of this study were Muslim Israelis of varying levels of religious observance, and different ages, socioeconomic status, and educational attainments. We chose snowball and convenience sampling as the research method, posting a call on social media for participants who would agree to answer an online questionnaire. Hence, the survey form was distributed by posting a link to an online Qualtrics form. The survey was available online for 3 months, from October to December 2021. The authors had no relationship with the participants prior to this study. Participants were informed that the research topic is: Medical uses of porcine-derived materials. The sole inclusion criterion was age: minimum age of 18, no minors. Responding to the ad were 809 Muslims. The demographic characteristics of the sample are presented in Table 1.

**Table 1:** Demographical descriptive data

Variable	Category	*N*	%	*M*	*SD*	*R*
*Gender*	Male	428	52.9			
	Female	381	47.1			
*Education*	Not academic	409	50.6			
	Academic	400	49.4			
*Religiosity*	Secular	427	52.8			
	Religious	382	47.2			
*Marital Status*	No relationship	74	9.1			
	In a relationship	735	90.9			
Age				47.72	20.59	18–81
Children				4.26	2.72	0–13

*Notes*. N = frequency. % = relative percent. M = mean. SD = standard deviation. R = range. Children = number of children

### Measures

The research questionnaire we constructed presents 15 types of medical treatments based on porcine-derived ingredients. We deliberately included different types of treatment, some of which are lifesaving and some are designed to relieve acute pain or prevent fatal diseases, while still others are elective and are not critical for saving life. Respondents were instructed to consider each medical treatment twice: the first time, to report their level of knowledge about to what extent each porcine-based treatment is permitted by Islam, on a 6-point Likert scale, with an additional optional answer “I do not know.“ The second time they were asked to express their opinion, using a 7-point Likert scale, to what degree they believe that their religion should permit each porcine-based treatment (See Appendix A for the study questionnaire). In this way we constructed two measures, by averaging the answers about the 15 medical treatments in terms of the respondent’s *Knowledge* about the permissibility of the use and in a similar manner, we averaged the answers to the 15 medical uses in terms of the respondent’s *Opinion* about whether each of the uses should be permitted.

### Ethical approval

The study was approved by the ethics review board of The Academic College of Tel-Aviv-Yaffo: Approval Number 2021 − 1001. Respondents signed informed consent forms to participate in the survey and to allow their data to be used by the researchers for statistical analyses; they also signed informed consent to allow the research team publish a scientific article based on their anonymous answers to the research questionnaire.

### Data analysis

Statistical analyses were conducted using the SPSS-PC (v26) statistical package. Prior to analysis of the data, data cleaning was performed and distribution characteristics, including tests of normality, were checked. Descriptive statistics were used to describe the sample characteristics. A path analysis model was designed to explain the respondents’ attitude to using porcine-based materials for medical purposes. Path analysis is a method of multiple regression statistical analysis that aims to evaluate causal models by examining the relationships between a dependent variable and two or more independent variables. This method enables the researcher to estimate both the magnitude and significance of causal connections between variables. Each path in a path analysis diagram graphically demonstrates a relationship between two variables and the range of the coefficients is -1 ≤ b ≤ 1. The significance level was set at p < .05.

## Results

**Fig. 1:**
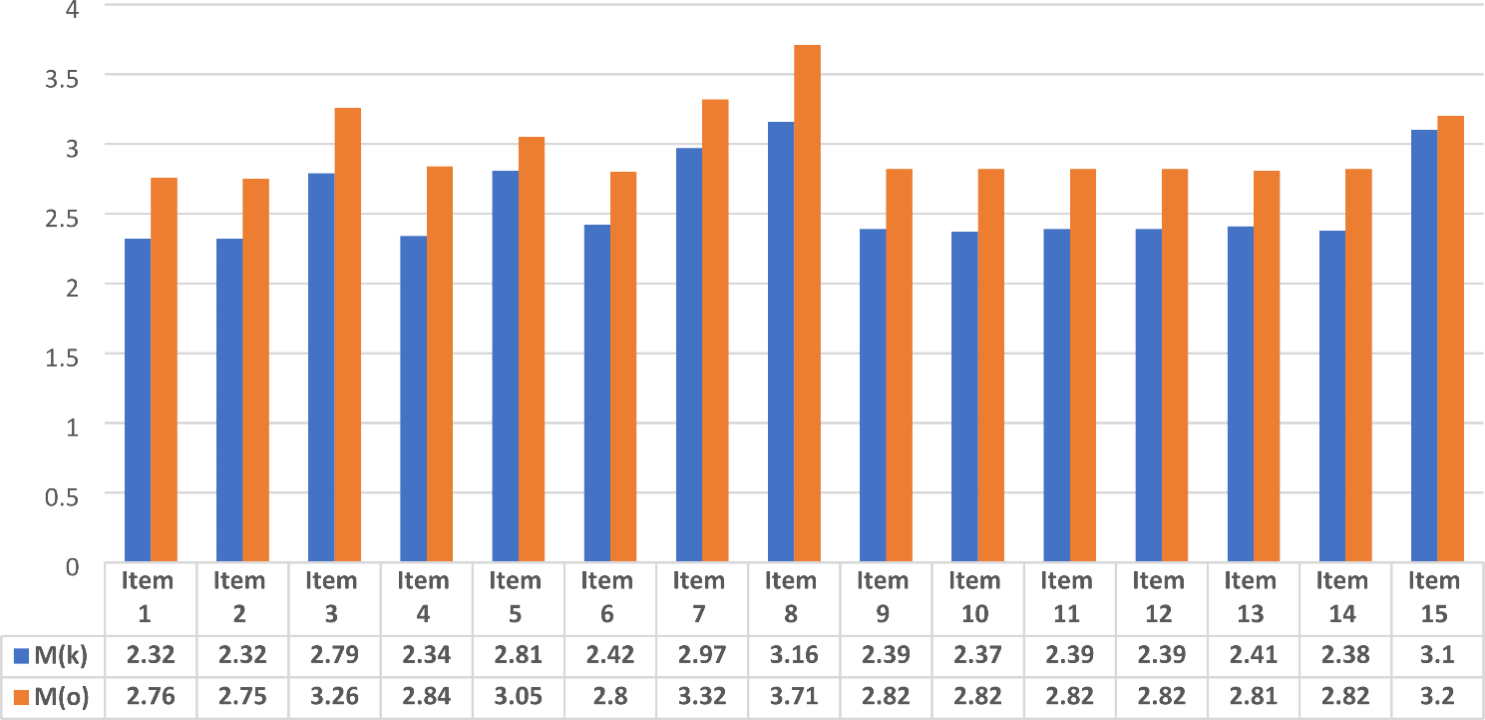
Bar graph for KNOWLEDGE and OPINION item means

*Notes*. M = mean. An indication of the letters *k* and *o* refer to the different measures, whereas *k* = KNOWLEDGE measure, and *o* = OPINION measure.

As previously mentioned, the *Knowledge* and the *Opinion* measures consist of 15 corresponding items each. Table 2 presents the means and standard deviations of each of the items and the zero-order correlations between each dyadic set of items. In addition, paired-samples *t*-tests were used to assess the degree of difference between 15 sets of items from both measures (i.e., item 1 in *Knowledge* vs. item 1 in *Opinion*, item 2 in *Knowledge* vs. item 2 in *Opinion* and so forth). Results of these t-tests show that for 12 out of the 15 treatments, the level of the opinion that supports the permissibility is greater than the level to which respondents believed these treatments are actually permitted. The means are also depicted in Fig. 1.

**Table 2:** Means, standard deviations, and Pearson zero-order correlations for all KNOWLEDGE and OPINION items

	KNOWLEDGE items		OPINION items			
Item	*M*	*SD*	*M*	*SD*	*r*_p_	*t*-test
1. Is it permissible according to the Muslim religion to cure a Muslim patient who is in danger of death from pancreatic insufficiency (due to cystic fibrosis) by administering drugs that are produced from a pig (such as pancreatic enzymes: amylase, lipase, protease)?	2.32	1.75	2.76	2.12	0.86	2.79^**^
2. Is it permissible according to the Muslim religion to prepare a vaccine for children against rotavirus, rubella, mumps, and measles made from pig proteins?	2.32	1.75	2.75	2.12	0.86	3.03^**^
3. Is it permissible according to the Muslim religion to transplant a valve taken from the heart of a pig to a Muslim patient suffering from a heart valve problem whose life is in danger?	2.79	1.77	3.26	2.07	0.92	1.67
4. Is it permissible according to the Muslim religion to give a Muslim patient suffering from severe chest pain painkillers that are produced from a pig?	2.34	1.76	2.84	2.14	0.88	0.13
5. Is it permissible according to the Muslim religion to implant in a Muslim patient cartilage for knees taken from a pig, for the purpose of replacing worn cartilage?	2.81	1.80	3.05	2.13	0.69	4.04^***^
6. Is it permissible according to the Muslim religion for a Muslim in training to use proteins produced from pigs for the purpose of building muscle as part of a training program at a gym?	2.42	1.87	2.80	2.17	0.91	3.99^***^
7. Is it permissible according to the Muslim religion to give a Muslim patient suffering from hypercoagulability (a tendency to form blood clots that may clog the blood vessels in the brain and heart) a blood thinner medicine produced from a pig?	2.97	1.83	3.32	2.15	0.73	1.63
8. Is it permissible according to the Muslim religion for Muslim researchers and scientists to practice on a pig in medical experiments or in invasive operations in order to improve medical treatments?	3.16	1.93	3.71	2.27	0.89	4.77^***^
9. Is it permissible according to the Muslim religion to give a Muslim patient albumin (protein) produced from a pig to maintain blood pressure levels in order to prevent a dangerous and drastic drop in blood pressure?	2.39	1.79	2.82	2.13	0.87	3.14^**^
10. Is it permissible according to the Muslim religion for a Muslim patient to wash their skin with soap made from pig fat in order to ameliorate a skin disease?	2.37	1.77	2.82	2.13	0.86	2.87^**^
11. Is it permissible according to the Muslim religion to make use of skin tissue from a pig for a Muslim patient for the purpose of skin graft after severe burns?	2.39	1.79	2.82	2.14	0.85	3.11^**^
12. Is it permissible according to the Muslim religion to give a Muslim baby suffering from cystic fibrosis fat-soluble vitamins -– E, A, K, D – that are produced from a pig?	2.39	1.78	2.82	2.14	0.84	3.24^**^
13. Is it permissible according to the Muslim religion to give a Muslim diabetic a medicine that is produced from a pig to lower their sugar/diabetic values?	2.41	1.80	2.81	2.13	0.85	3.52^***^
14. Is it permissible according to the Muslim religion to give a pregnant Muslim woman a steroid drug produced from a pig, in order to accelerate fetal lung maturation in a situation where the woman has preterm labor contractions in the seventh month?	2.38	1.79	2.82	2.14	0.84	2.68^**^
15. Is it permissible according to the Muslim religion to give a Muslim preterm infant who was born in the seventh month and suffers from respiratory distress a medicine produced from a pig?	3.10	1.85	3.20	2.20	0.61	5.81^***^

*Notes*. **p < .01, ***p < .001. M = mean. SD = standard deviation. r = correlation coefficient (all correlations are significant at *p* < .001). Generally, the internal consistencies, as measured by Cronbach’s Alpha reliability coefficients, of the KNOWLEDGE and OPINION variables are 0.98 and 0.99, respectively

**Fig. 2:**
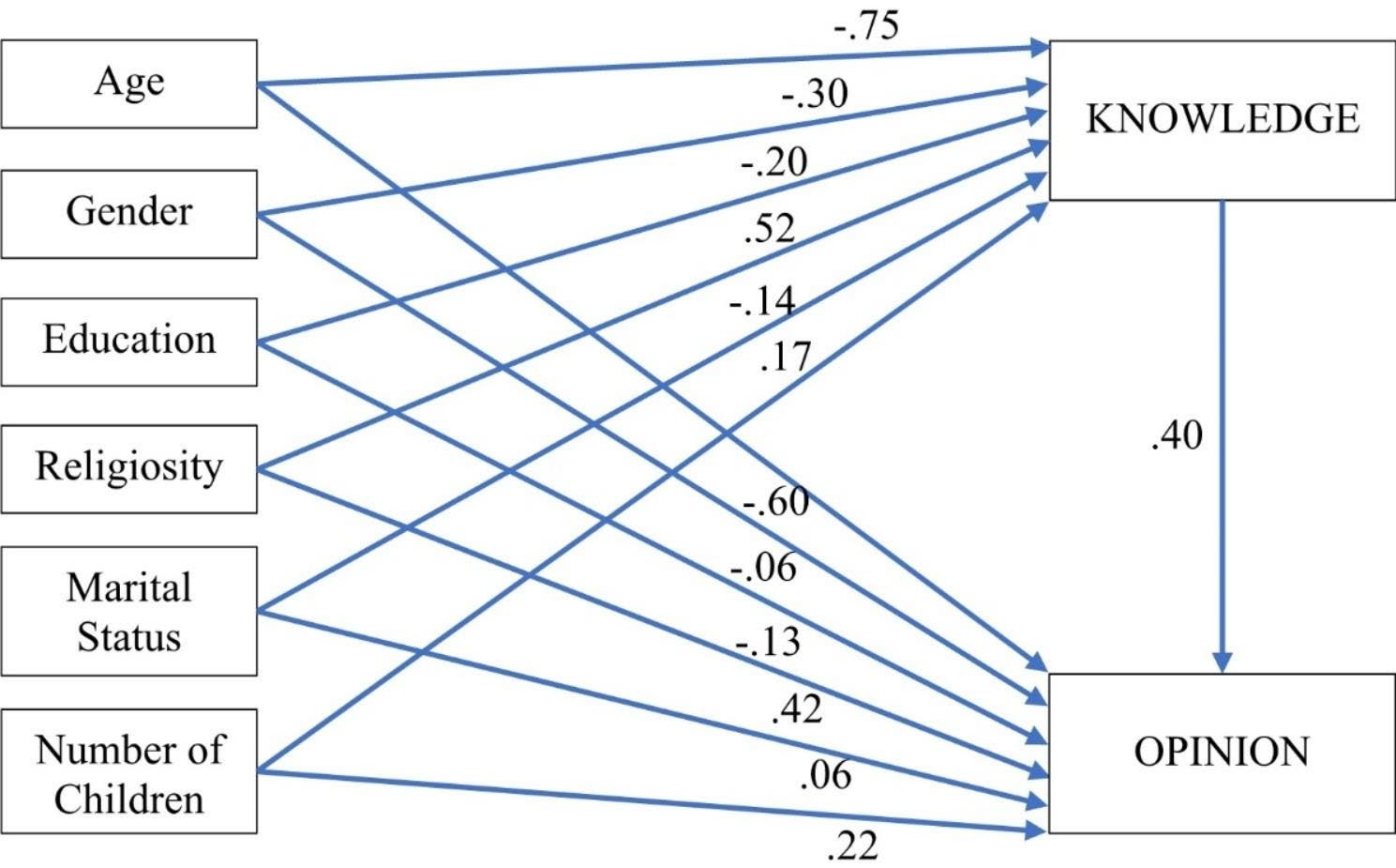
Mediation path model diagram with standardized regression coefficients. *Notes*. All path coefficients are significant at *p* < .001. Gender: 0 = female, 1 = male. Education: 0 = not academic, 1 = academic. Religiosity: 0 = secular, 1 = religious. Marital status: 0 = no relationship, 1 = in a relationship

In addition, path analysis and SEM (structural equation modeling) were employed to test a mediation model, in which the demographical parameters act as predictors, the *Knowledge* is the mediator, and the *Opinion* is the criterion. Negative coefficients of paths represent negative connections, and positive coefficients of paths represent positive connections, similarly to all linear regression statistics. The larger the absolute value of a path coefficient, the more intense is the connection. All fit measures of the model indicate an absolute fit [40] χ^2^ (6) = 7.52, *p* = .275, χ^2^/df = 1.25, SRMR = 0.02, CFI = 0.99, GFI = 0.99, NFI = 0.98, TLI = 0.97, RMSEA (90% CI) = 0.03 (0.00-0.05), *p-close* = 0.941. In addition, the path analysis used bootstrapping to assess the mediation effect (5,000 resamples, 95% bias-corrected confidence interval). The results of the analyses are depicted in Table 3; Fig. 2. Results show that all the path coefficients are significant, for both the indirect and the direct connections between the socio-demographic variables and the Knowledge as well as the Opinion variables. Respondents who are younger, female, less educated, more religious, in a marital relationship, and have more children tended to believe that the use of porcine materials for medical purposes is permitted by Islam. As to respondents’ opinion regarding the permissibility of such use, younger individuals with lower educational level, more religious, and those who have more children believed that porcine derived medical treatments should be permissible. The more the respondents believed these treatments are religiously permitted, the more they tended to support the view that such uses should be permissible. The knowledge that respondents possessed on the issue of permissibility mediates the associations between socio-demographic variables (Age, Gender, Education, Religiosity, Marital Status, Number of Children) and their opinion on the topic.

**Table 3:** Path analysis results with standardized regression coefficients and difference tests

Path			β	*SE*	*t-test*	*Sig.*
Age	→	KNOWLEDGE	− 0.75	0.00	-15.10	0.000
Gender	→	KNOWLEDGE	− 0.30	0.07	-13.68	0.000
Education	→	KNOWLEDGE	− 0.20	0.08	-8.31	0.000
Religiosity	→	KNOWLEDGE	0.52	0.11	16.36	0.000
Marital Status	→	KNOWLEDGE	− 0.14	0.15	5.64	0.000
Children	→	KNOWLEDGE	0.17	0.04	2.95	0.003
Age	→	OPINION	− 0.60	0.00	-15.58	0.000
Gender	→	OPINION	− 0.06	0.07	-3.52	0.001
Education	→	OPINION	− 0.13	0.07	-7.85	0.000
Religiosity	→	OPINION	0.42	0.10	17.02	0.000
Marital Status	→	OPINION	0.06	0.12	3.52	0.010
Children	→	OPINION	0.22	0.03	5.50	0.000
KNOWLEDGE	→	OPINION	0.40	0.03	16.63	0.000

**Table:** (Continued Table 3)

Path					Effect	LL	UL	*Sig.*
Age	→	KNOWLEDGE	→	OPINION	− 0.30	− 0.36	− 0.24	0.000
Gender	→	KNOWLEDGE	→	OPINION	− 0.12	− 0.14	− 0.10	0.000
Education	→	KNOWLEDGE	→	OPINION	− 0.08	− 0.10	− 0.06	0.000
Religiosity	→	KNOWLEDGE	→	OPINION	0.20	0.17	0.25	0.000
Marital Status	→	KNOWLEDGE	→	OPINION	− 0.06	− 0.08	− 0.04	0.000
Children	→	KNOWLEDGE	→	OPINION	0.07	0.02	0.11	0.002

*Notes*. SE = standard error. Children = number of children. Gender: 0 = female, 1 = male. Education: 0 = not academic, 1 = academic. Religiosity: 0 = secular, 1 = religious. Marital status: 0 = no relationship, 1 = in a relationship. Analyses used bootstrapping (95% bias−corrected, 5,000 resamples). Effect = standardized indirect effect (predictor→through mediator→criterion). LL = lower limit of the confidence interval; UL = upper limit of the confidence interval. Children = number of children. Gender: 0 = female, 1 = male. Education: 0 = not academic, 1 = academic. Religiosity: 0 = secular, 1 = religious. Marital status: 0 = no relationship, 1 = in a relationship

Figure 2 graphically illustrates the direct and the indirect causal associations between the variables of the mediation model. Each socio-demographic variable predicts the opinion regarding the permissibility, in both a direct as well as an indirect association.

## Discussion

The current study is the first comprehensive and wide-ranging project that examined to what extent Muslims believe that their religion permits medical treatments based on porcine-derived substances, and to what extent they believe these treatments should be allowed according to religious laws and rulings. It supplements the scant previous research on this topic that documented the devout obedience of Muslim patients to their religious laws [3, 4, 6–8, 11, 12, 17]. The contribution of the present study is threefold. First, this is a pioneering empirical study that included a wide range of uses of pigs, including the production of vaccines and vitamins, skin grafts and treatments, in addition to drugs and transplants. Second, thanks to the relatively large number of participants of the Muslim faith, the second largest major religion group in the world, a comparison was made between the level of knowledge and the attitudes of Muslims regarding the use of pigs for a wide range of medical needs.

In addition to measuring the attitude toward using porcine-derived treatments that had been conducted in previous studies, [2, 3, 11–13] this is the first empirical study that evaluated the level to which Muslims believe their religion permits these treatments. Our findings indicate that most respondents are not familiar with the permissibility of using pigs in *darrurah* situations, and accordingly, they express negative attitudes about allowing it. Muslims tend to comply with religious directives in all areas of life, including medical problems [42, 43]. The level of *Knowledge* of the individual as to whether their religion forbids or permits it according to Shariah served as a variable that explains their position toward the use of pigs. When we asked about each respondent’s knowledge as to whether porcine materials are allowed by their religion for each of the 15 different uses, about 20% of respondents answered for each of these uses that they do not know the answer.

The inclination to obey religious rulings is manifested in the positive beta coefficients in the path analysis: the more the respondent believed a certain porcine-derived medical use is permitted, the higher is their tendency to support this treatment. These positive associations imply that the *Opinion* of Muslims regarding the permissibility of using porcine-derived materials draws on their layman’s understanding of what is permitted and what is prohibited by the Shariah. We therefore suggest that inaccurate knowledge and limited awareness reduce the tendency to accept these treatments.

Third, the findings pave the way for formulating several important implications for practice as follows:

The attending medical staff must work according to the code of ethics that guarantees the patient’s right to receive complete, relevant, and accessible information about the source of the materials used for medical treatment, in order to fulfill the health, emotional, spiritual, cultural, and religious needs of Muslim patients. Informing patients about this issue demonstrates respect for their religious beliefs and may promote the therapeutic alliance; therefore, this could have implications for the public health in improving the trust that Muslim patients have in their physicians.

Providing information about the available alternative therapies is part of informed consent and constitutes part of the respect for the patient’s autonomy. Recognition of the religious beliefs of the Muslim patient is a very important factor in the interaction between physician and patient to obtain informed consent for medical treatment [5, 39]. Failure to respect religious sensitivities regarding the use of biological products such as materials from porcine sources can have serious consequences. Physicians, pharmacists, nurses, and transplant surgeons have a profound responsibility to be aware and sensitive to the patient’s religious background [42, 43] For example, information on the gelatin and stearic acid content of drugs should be provided and, if possible, alternatives should be considered. When no substitute can be used, patients, family members, guardians, and even religious leaders may be involved in the decision-making process [7, 8]. It is highly recommended for hospitals to engage their local Muslim communities to create general guidelines and policies for that hospital so that those Muslims who do not agree with those guidelines or policies can take their patients to a different hospital if possible, and thus make timely and informed decisions [37–39].

The medical staff is obligated to provide patients with sufficient information to enable them to make an informed judgment about undergoing treatment, including medications or transplantation of an organ from a pig [44]. It is very important for healthcare providers to keep themselves up to date with knowledge of the subject in order to provide all appropriate information that is required to fulfill the criteria of a valid informed consent. Medical councils should consider introducing a new practice of adding a separate consent form for the biological implant or device; alternatively, an additional clause focusing on the animal source of the treatment may supplement the existing informed consent forms. This will not only protect doctors and patients but also will bring the informed consent in line with the Good Medical Practice. It is important to involve the Muslim patient and their relatives in the decision making related to their medical care. They may wish to consult with their cleric or with the hospital chaplain to fully ensure that the animal-derived surgical product is acceptable for healing purposes when no other available alternative exists [38–40].

### Study limitations

Along with the contributions and benefits of this study, we must acknowledge potential limitations. One of them relates to the fact that an accurate assessment of attitudes towards advanced medical treatments, as described in our questionnaire, may be too complex to be evaluated by a conventional quantitative research methodology [45, 46]. Another limitation concerns the self-selection bias that usually appears in volunteer sampling, where respondents who agreed to take part in the study may have different tendencies and attitudes towards the research topic than those who chose not to participate. Likewise, the representativeness of the sample is also questionable since no random sampling was performed but rather a volunteer sampling.

## Conclusion

It is important to consider the religious beliefs of all patients, and their choice of treatment should be respected. Muslim patients and various religious leaders might have different views regarding the use of porcine-derived products for saving life. It is necessary to obtain full informed consent for the use of porcine-derived products for followers of Islam, since they may oppose the treatment due to lack of proper knowledge. The preferences of these patients may require physicians to take a proactive approach and initiate explanations rather than passively waiting for patients to ask them about this issue. We hope that the findings of the research project will serve as an empirical basis for future discussions between physicians, ethicists, religious leaders, and theologians to promote cultural competence among the medical and nursing staff as well as respect for the autonomy of Muslim patients.

### Electronic supplementary material

Below is the link to the electronic supplementary material.

Supplementary Material 1

## Data Availability

The data of this research project can be obtained from the corresponding author upon reasonable request.
