# Supplementary material for: The muslim patient and medical treatments based on porcine ingredients
Source: BMC Med Ethics. 2023 Oct 27;24:89. doi: 10.1186/s12910-023-00975-0 (PMC10612269; doi:10.1186/s12910-023-00975-0)
Supplement: Supplementary file 1 — Supplementary Material 1 [file 12910_2023_975_MOESM1_ESM.docx]

**Appendix A: The research questionnaire**

1- **Age** (years):

2- **Gender**: 1 - Male 2- Female

3- **Religion**: 1. Muslim 2. Jew

4- **Education**: 1. Elementary 2. High school 3. Academic

5- **Level of religiosity**: 1- Religious, 2- Traditional, 3- Secular

6- **Family status**: 1. In a relationship 2. Not in a relationship

The first part of the questionnaire presents you with a number of medical treatment scenarios. Please indicate, to the best of your knowledge, to what extent the Muslim religion allows medical treatment as described in the scenario:

1. Is it permissible according to the Muslim religion to cure a Muslim patient who is in danger of death from pancreatic insufficiency (due to cystic fibrosis) by administering drugs that are produced from a pig (such as pancreatic enzymes: amylase, lipase, protease)?

| 1. It is 100% forbidden | 1. It is almost always forbidden | 1. 3 It is permitted infrequently |  | 4  It is permitted in exceptional cases | 5  It is always almost permitted | 6  It is totally  100%  permitted ` | 9  I don’t know |
| --- | --- | --- | --- | --- | --- | --- | --- |

2. Is it permissible according to the Muslim religion to prepare a vaccine for children against rotavirus, rubella, mumps, and measles made from pig proteins?

| 1 It is 100% forbidden | 2 It is almost always forbidden | 1. 3 It is permitted infrequently |  | 4  It is permitted in exceptional cases | 5  It is always almost permitted | 6  It is totally  100%  permitted | 9  I don’t know |
| --- | --- | --- | --- | --- | --- | --- | --- |

3. Is it permissible according to the Muslim religion to transplant a valve taken from the heart of a pig to a Muslim patient suffering from a heart valve problem whose life is in danger?

| 1 It is 100% forbidden | 2 It is almost always forbidden | 1. 3 It is permitted infrequently |  | 4  It is permitted in exceptional cases | 5  It is always almost permitted | 6  It is totally  100%  permitted ` | 9  I don’t know |
| --- | --- | --- | --- | --- | --- | --- | --- |

4. Is it permissible according to the Muslim religion to give a Muslim patient suffering from severe chest pain painkillers that are produced from a pig?

| 1 It is 100% forbidden | 2 It is almost always forbidden | 1. 3 It is permitted infrequently |  | 4  It is permitted in exceptional cases | 5  It is always almost permitted | 6  It is totally  100%  permitted ` | 9  I don’t know |
| --- | --- | --- | --- | --- | --- | --- | --- |

5. Is it permissible according to the Muslim religion to implant in a Muslim patient cartilage for knees taken from a pig, for the purpose of replacing worn cartilage?

| 1 It is 100% forbidden | 2 It is almost always forbidden | 1. 3 It is permitted infrequently |  | 4  It is permitted in exceptional cases | 5  It is always almost permitted | 6  It is totally  100%  permitted ` | 9  I don’t know |
| --- | --- | --- | --- | --- | --- | --- | --- |

6. Is it permissible according to the Muslim religion for a Jew in training to use proteins produced from pigs for the purpose of building muscle as part of a training program at a gym?

| 1 It is 100% forbidden | 2 It is almost always forbidden | 1. 3 It is permitted infrequently |  | 4  It is permitted in exceptional cases | 5  It is always almost permitted | 6  It is totally  100%  permitted ` | 9  I don’t know |
| --- | --- | --- | --- | --- | --- | --- | --- |

7. Is it permissible according to the Muslim religion to give a Muslim patient suffering from hypercoagulability (a tendency to form blood clots that may clog the blood vessels in the brain and heart) a blood thinner medicine produced from a pig?

| 1 It is 100% forbidden | 2 It is almost always forbidden | 1. 3 It is permitted infrequently |  | 4  It is permitted in exceptional cases | 5  It is always almost permitted | 6  It is totally  100%  permitted ` | 9  I don’t know |
| --- | --- | --- | --- | --- | --- | --- | --- |

8. Is it permissible according to the Muslim religion for Muslim researchers and scientists to practice on a pig in medical experiments or in invasive operations in order to improve medical treatments?

| 1 It is 100% forbidden | 2 It is almost always forbidden | 1. 3 It is permitted infrequently |  | 4  It is permitted in exceptional cases | 5  It is always almost permitted | 6  It is totally  100%  permitted ` | 9  I don’t know |
| --- | --- | --- | --- | --- | --- | --- | --- |

9. Is it permissible according to the Muslim religion to give a Muslim patient albumin (protein) produced from a pig to maintain blood pressure levels in order to prevent a dangerous and drastic drop in blood pressure?

| 1 It is 100% forbidden | 2 It is almost always forbidden | 1. 3 It is permitted infrequently |  | 4  It is permitted in exceptional cases | 5  It is always almost permitted | 6  It is totally  100%  permitted ` | 9  I don’t know |
| --- | --- | --- | --- | --- | --- | --- | --- |

10. Is it permissible according to the Muslim religion for a Muslim patient to wash their skin with soap made from pig fat in order to ameliorate a skin disease?

| 1 It is 100% forbidden | 2 It is almost always forbidden | 1. 3 It is permitted infrequently |  | 4  It is permitted in exceptional cases | 5  It is always almost permitted | 6  It is totally  100%  permitted ` | 9  I don’t know |
| --- | --- | --- | --- | --- | --- | --- | --- |

11. Is it permissible according to the Muslim religion to make use of skin tissue from a pig for a Muslim patient for the purpose of skin graft after severe burns?

| 1 It is 100% forbidden | 2 It is almost always forbidden | 1. 3 It is permitted infrequently |  | 4  It is permitted in exceptional cases | 5  It is always almost permitted | 6  It is totally  100%  permitted ` | 9  I don’t know |
| --- | --- | --- | --- | --- | --- | --- | --- |

12. Is it permissible according to the Muslim religion to give a Muslim baby suffering from cystic fibrosis fat-soluble vitamins -– E, A, K, D – that are produced from a pig?

| 1 It is 100% forbidden | 2 It is almost always forbidden | 1. 3 It is permitted infrequently |  | 4  It is permitted in exceptional cases | 5  It is always almost permitted | 6  It is totally  100%  permitted ` | 9  I don’t know |
| --- | --- | --- | --- | --- | --- | --- | --- |

13. Is it permissible according to the Muslim religion to give a Muslim diabetic a medicine that is ​​produced from a pig to lower their sugar values?

| 1 It is 100% forbidden | 2 It is almost always forbidden | 1. 3 It is permitted infrequently |  | 4  It is permitted in exceptional cases | 5  It is always almost permitted | 6  It is totally  100%  permitted ` | 9  I don’t know |
| --- | --- | --- | --- | --- | --- | --- | --- |

14. Is it permissible according to the Muslim religion to give a pregnant Muslim woman a steroid drug produced from a pig, in order to accelerate fetal lung maturation in a situation where the woman has preterm labor contractions in the seventh month?

| 1 It is 100% forbidden | 2 It is almost always forbidden | 1. 3 It is permitted infrequently |  | 4  It is permitted in exceptional cases | 5  It is always almost permitted | 6  It is totally  100%  permitted ` | 9  I don’t know |
| --- | --- | --- | --- | --- | --- | --- | --- |

15. Is it permissible according to the Muslim religion to give a Muslim preterm infant who was born in the seventh month and suffers from respiratory distress a medicine produced from a pig?

| 1 It is 100% forbidden | 2 It is almost always forbidden | 1. 3 It is permitted infrequently |  | 4  It is permitted in exceptional cases | 5  It is always almost permitted | 6  It is totally  100%  permitted ` | 9  I don’t know |
| --- | --- | --- | --- | --- | --- | --- | --- |

In the second part of the questionnaire, you are asked to read each scenario and state your personal opinion on whether and to what extent you support providing the medical treatment described in the scenario.

16. Do you think that treatment should be approved for a Muslim patient whose life is in danger due to pancreatic insufficiency (due to cystic fibrosis) by administering drugs that are produced from a pig (such as pancreatic enzymes: amylase, lipase, protease)?

| 1  Totally disagree | 2  Disagree somewhat | 3  Agree slightly | 4  Agree moderately | 5  Agree to a large extent | 6  Agree strongly | 7  Totally agree |
| --- | --- | --- | --- | --- | --- | --- |

17. Do you think that vaccinating Muslim children against rotavirus, rubella, mumps, and measles using a vaccine produced from a pig should be allowed ?

| 1  Totally disagree | 2  Disagree somewhat | 3  Agree slightly | 4  Agree moderately | 5  Agree to a large extent | 6  Agree strongly | 7  Totally agree |
| --- | --- | --- | --- | --- | --- | --- |

18. Do you think that a Muslim heart patient should be allowed to have a transplant of a heart valve taken from a pig?

| 1  Totally disagree | 2  Disagree somewhat | 3  Agree slightly | 4  Agree moderately | 5  Agree to a large extent | 6  Agree strongly | 7  Totally agree |
| --- | --- | --- | --- | --- | --- | --- |

19. Do you think that a Muslim patient should be allowed to take medicine produced from a pig for the purpose of relieving angina pain?

| 1  Totally disagree | 2  Disagree somewhat | 3  Agree slightly | 4  Agree moderately | 5  Agree to a large extent | 6  Agree strongly | 7  Totally agree |
| --- | --- | --- | --- | --- | --- | --- |

20. Do you think a Muslim patient should be allowed to receive a knee cartilage transplant produced from a pig for the purpose of replacing worn cartilage?

| 1  Totally disagree | 2  Disagree somewhat | 3  Agree slightly | 4  Agree moderately | 5  Agree to a large extent | 6  Agree strongly | 7  Totally agree |
| --- | --- | --- | --- | --- | --- | --- |

21. Do you think that a Jew in training should be allowed to use muscle-building protein products that are produced from a pig as part of a training program at a gym?

| 1  Totally disagree | 2  Disagree somewhat | 3  Agree slightly | 4  Agree moderately | 5  Agree to a large extent | 6  Agree strongly | 7  Totally agree |
| --- | --- | --- | --- | --- | --- | --- |

22. Do you think that a Muslim patient who suffers from excessive blood clotting (tendency to form blood clots that can clog the blood vessels in the brain and heart) should be allowed to be treated with a blood-thinning drug that is produced from a pig?

| 1  Totally disagree | 2  Disagree somewhat | 3  Agree slightly | 4  Agree moderately | 5  Agree to a large extent | 6  Agree strongly | 7  Totally agree |
| --- | --- | --- | --- | --- | --- | --- |

23. Do you think that Muslim researchers and scientists should be allowed to use the pig in experiments or invasive procedures to practice on to improve treatments?

| 1  Totally disagree | 2  Disagree somewhat | 3  Agree slightly | 4  Agree moderately | 5  Agree to a large extent | 6  Agree strongly | 7  Totally agree |
| --- | --- | --- | --- | --- | --- | --- |

24. Do you think that a Muslim patient should be allowed to be given albumin - a protein produced from a pig that restores the maintenance of blood pressure to prevent a dangerous and drastic drop in blood pressure?

| 1  Totally disagree | 2  Disagree somewhat | 3  Agree slightly | 4  Agree moderately | 5  Agree to a large extent | 6  Agree strongly | 7  Totally agree |
| --- | --- | --- | --- | --- | --- | --- |

25. Do you think that a Muslim patient should be allowed to use medical soap made from pig fat to heal a skin disease?

| 1  Totally disagree | 2  Disagree somewhat | 3  Agree slightly | 4  Agree moderately | 5  Agree to a large extent | 6  Agree strongly | 7  Totally agree |
| --- | --- | --- | --- | --- | --- | --- |

26. Do you think a Muslim patient should be allowed to use tissue from a pig for skin grafting after severe burns?

| 1  Totally disagree | 2  Disagree somewhat | 3  Agree slightly | 4  Agree moderately | 5  Agree to a large extent | 6  Agree strongly | 7  Totally agree |
| --- | --- | --- | --- | --- | --- | --- |

27. Do you think it should be permitted to give a Muslim baby who is sick with cystic fibrosis fat-soluble vitamins E, A, K D that are produced from a pig?

| 1  Totally disagree | 2  Disagree somewhat | 3  Agree slightly | 4  Agree moderately | 5  Agree to a large extent | 6  Agree strongly | 7  Totally agree |
| --- | --- | --- | --- | --- | --- | --- |

28. Do you think a Muslim diabetic should be allowed to take a drug produced from a pig to lower sugar values ​​?

| 1  Totally disagree | 2  Disagree somewhat | 3  Agree slightly | 4  Agree moderately | 5  Agree to a large extent | 6  Agree strongly | 7  Totally agree |
| --- | --- | --- | --- | --- | --- | --- |

29. Do you think that a Muslim pregnant woman should be allowed to be given a steroid drug produced from a pig, in order to speed up fetal lung maturation in a situation where the woman has preterm labor contractions in the seventh month?

| 1  Totally disagree | 2  Disagree somewhat | 3  Agree slightly | 4  Agree moderately | 5  Agree to a large extent | 6  Agree strongly | 7  Totally agree |
| --- | --- | --- | --- | --- | --- | --- |

30. Do you think that a Muslim preterm infant who was born at week 33 and suffers from respiratory distress should be allowed to be given a drug produced from a pig?

| 1  Totally disagree | 2  Disagree somewhat | 3  Agree slightly | 4  Agree moderately | 5  Agree to a large extent | 6  Agree strongly | 7  Totally agree |
| --- | --- | --- | --- | --- | --- | --- |
